# A prospective study on endoscopic ultrasound for the differential diagnosis of serous cystic neoplasms and mucinous cystic neoplasms

**DOI:** 10.1186/s12876-019-1035-8

**Published:** 2019-07-16

**Authors:** Lisen Zhong, Ningli Chai, Enqiang Linghu, Huikai Li, Jing Yang, Ping Tang

**Affiliations:** Department of Gastroenterology and Hepatology, Chinese PLA General Hospital, Fuxing Road 28, Haidian District, Beijing, 100853 China

**Keywords:** Endoscopic ultrasound, Serous cystic neoplasm, Mucinous cystic neoplasm, Pancreatic cystic neoplasm, Diagnosis

## Abstract

**Background:**

To provide criteria for the differential diagnosis of serous cystic neoplasms (SCNs) and mucinous cystic neoplasms (MCNs) by analyzing the imaging features of these two neoplasms by endoscopic ultrasound (EUS).

**Methods:**

From April 2015 to December 2017, a total of 69 patients were enrolled in this study. All patients were confirmed to have MCNs (31 patients) or SCNs (38 patients) by surgical pathology. All patients underwent EUS examination. The observation and recorded items were size, location, shape, cystic wall thickness, number of septa, and solid components.

**Results:**

Head/neck location, lobulated shape, thin wall and > 2 septa were the specific imaging features for the diagnosis of SCNs. When any two imaging features were combined, we achieved the highest area under the curve (Az) (0.824), as well as the appropriate sensitivity (84.2%), specificity (80.6%), positive predictive value (PPV) (84.2%), and negative predictive value (NPV) (80.6%). Body/tail location, round shape, thick wall and 0–2 septa were the specific imaging features for the diagnosis of MCNs. When any three imaging features were combined, we obtained the highest Az value (0.808), as well as the appropriate sensitivity (77.4%), specificity (84.2%), PPV (80.0%) and NPV (82.1%).

**Conclusions:**

Pancreatic cystadenomas that meet any two of the four imaging features of head/neck location, lobulated shape, thin wall and > 2 septa could be diagnosed as SCNs, and those that meet any three of the four imaging features of body/tail location, round shape, thick wall and 0–2 septa could be considered as MCNs.

**Trial registration:**

The study was registered at the Chinese Clinical Trial Registry. The registration identification number is ChiCTR-OOC-15006118. The date of registration is 2015-03-20.

## Introduction

Pancreatic cystic neoplasms (PCNs) mainly include serous cystic neoplasms (SCNs) and mucinous cystic neoplasms (MCNs), accounting for 10 to 15% of pancreatic cystic lesions (PCLs), and 1 to 2% of pancreatic tumors [1]. Because of its deep location, slow growth and no clinical symptoms in the early stage, PCNs are easily misdiagnosed [2]. SCNs and MCNs have different biological behaviors. Relevant studies have reported that only 1 to 3% of SCNs have been transformed into serous cystadenocarcinomas [3]. To date, only 25–30 serous cystadenocarcinomas have been reported worldwide [4]. Therefore, SCNs are generally considered benign and can be followed up [5]. MCNs have malignant potential and are recommended for surgical resection once an adequate diagnosis has been performed [6, 7]. Therefore, it is crucial to accurately differentiate between SCNs and MCNs for the appropriate treatment.

Computed tomography (CT) and magnetic resonance imaging (MRI) are routine abdominal examinations in China that can effectively screen pancreatic masses. However, due to the limited resolution, these two imaging modalities do not accurately and effectively present the microstructure of PCLs, which increases the difficulty of differential diagnosis of SCNs and MCNs. However, due to its high spatial resolution, endoscopic ultrasound (EUS) can effectively reveal the microstructure of PCLs (such as the septa and thickness of the cystic wall), which greatly improves the diagnostic accuracy of PCLs [8]. At present, many studies have demonstrated the imaging features of SCNs and MCNs [9, 10], but these studies do not generate effective diagnostic criteria. The purpose of our study is to provide criteria for the differential diagnosis of SCNs and MCNs by analyzing the imaging features of these two neoplasms by EUS.

## Methods

This study was approved by the Ethics Committee of the Chinese People’s Liberation Army General Hospital.

### Patients

From April 2015 to December 2017, we prospectively enrolled 88 patients with PCNs who underwent EUS and ultimately received surgery at the Chinese PLA General Hospital. Among these patients, 69 were proven to have MCNs (31 patients) or SCNs (38 patients) by surgical pathology. All patients had neither contraindications to EUS examination nor a history of acute pancreatitis and pancreatic necrosis. All of patients signed informed consent forms.

### EUS examination

Before the examination, the patient fasted for at least 8 h. In our study, we used an ultrasonic endoscope (GF-UCT260; Olympus, Tokyo, Japan) in the procedures. To ensure the imaging quality, an echoprobe was routinely covered with a water-filled balloon. During the procedures, the patients were under general anesthesia. The examination was performed by endoscopic physicians with at least 5 years of experience. The EUS findings were recorded in the form of video or picture.

### Imaging analysis

Two endoscopic physicians with more than at least 5 years of experience independently completed the analysis of EUS images without knowing the clinical data, other imaging findings and pathological diagnosis of the patients. The final conclusion was drawn after the endoscopic physicians discussed the results and came to an agreement if there was dissent. The observation and recorded items were: size (the longest axis), location (head/neck or body/tail), shape (round, lobulated, and irregular), cystic wall thickness (0–2 mm and > 2 mm, the thickest part of cystic wall was considered thick if it was > 2 mm and thin if it was 2 mm or less), number of septa (0–2 and > 2), and solid components (solid tissues, such as mural nodules, except septa in cystic lesions).

### Statistical analysis

SPSS 17 statistical software was used for statistical analysis. The measurement data were presented as the mean ± SD and tested by the t-test. The count data was tested by the chi-square test or continuity correction. Interobserver agreement was assessed by Kappa statistics. The agreement was graded as follows: poor (0.01–0.20), moderate (0.21–0.40), fair (0.41–0.60), good (0.61–0.80), or excellent (0.81–1.00). The sensitivity, specificity, positive predictive value (PPV), negative predictive value (NPV) and area under the receiver operating characteristic curves (Az) were used to analyze the efficacy of different imaging features for the differential diagnosis of SCNs and MCNs. The difference was considered statistically significant at *P* < 0.05.

## Results

### Basic characteristics of the patients

As shown in Table 1, a total of 69 patients were enrolled in this study. Thirty-eight patients (8 males, 30 females) were confirmed to have SCNs by surgical pathology, with an average age of 49.16 ± 14.52 (range, 18–77) years. Thirty-one patients (4 males, 27 females) were confirmed to have MCNs by surgical pathology, with an average age of 46.39 ± 13.30 (range, 19–69) years. There was no significant difference in age and sex between SCNs and MCNs (*P* = 0.277).

**Table 1:** Basic characteristics of the 69 patients enrolled

	SCNs	MCNs	*P*
Sex(male/female)	8/30	4/27	0.374
Age, mean ± SD, yr	49.16 ± 13.30	46.39 ± 13.30	0.416

*SCNs* serous cystic neoplasms, *MCNs* mucinous cystic neoplasms

### Imaging features

The comparison of imaging features between SCNs and MCNs is shown in Table 2.

**Table 2:** Comparison of imaging features between SCNs and MCNs

Imaging features	SCNs	MCNs	*P*
Size (cm), mean ± SD	4.53 ± 2.34	4.90 ± 2.38	0.520
Location			
Head/neck	18 (47.4%)	5 (16.1%)	0.006
Body/tail	20 (52.6%)	26 (83.9%)	
Shape			0.000
Round	10 (26.3%)	24 (77.4%)	
Lobulated	21 (55.3%)	4 (12.9%)	
Irregular	7 (18.4%)	3 (9.7%)	
Wall thickness			0.000
Thin (0–2 mm)	29 (76.3%)	10 (32.3%)	
Thick (> 2 mm)	9 (23.7%)	21 (67.7%)	
Number of septa			0.003
0–2 septa	11 (28.9%)	20 (64.5%)	
>2 septa	27 (71.1%)	11 (35.5%)	
Solid components			0.168
Positive	3 (7.9%)	7 (22.6%)	
Negative	35 (92.1%)	24 (77.4%)	

*SCNs* serous cystic neoplasms, *MCNs* mucinous cystic neoplasms

**Fig. 1:**
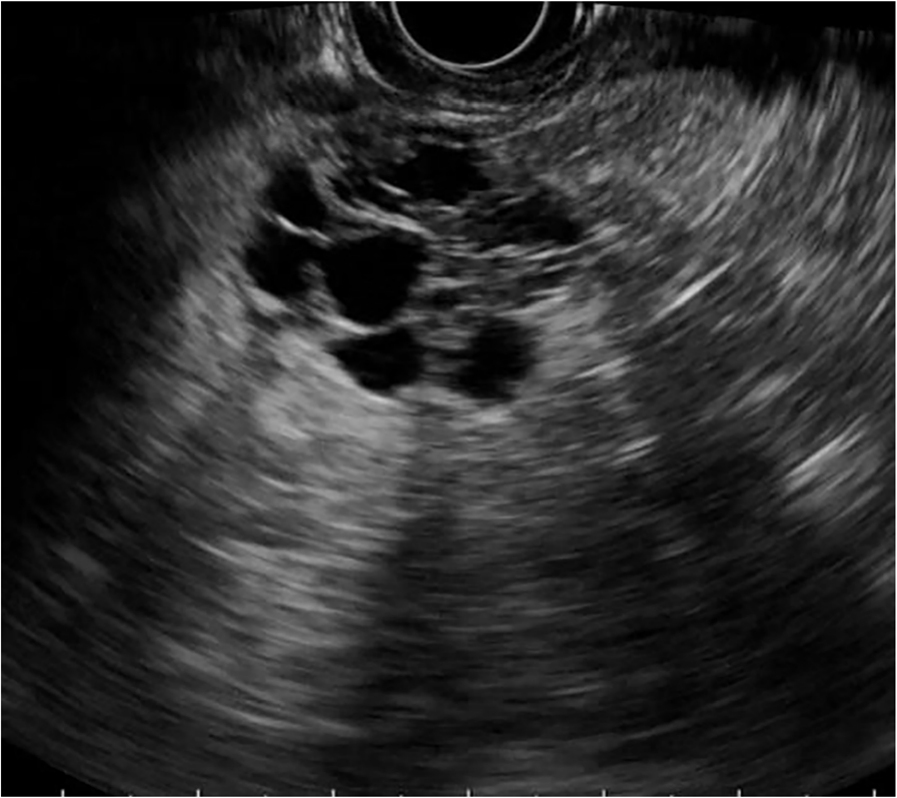
Serous cystic neoplasm with lobulated shape and multiple septa

**Fig. 2:**
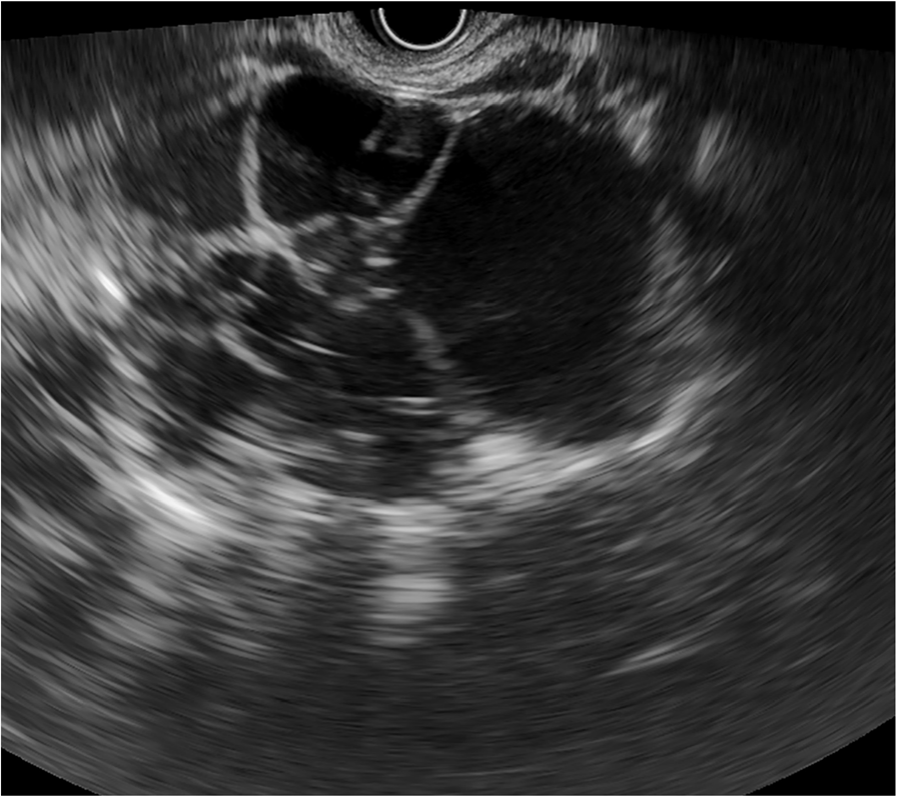
Serous cystic neoplasm with irregular shape

**Fig. 3:**
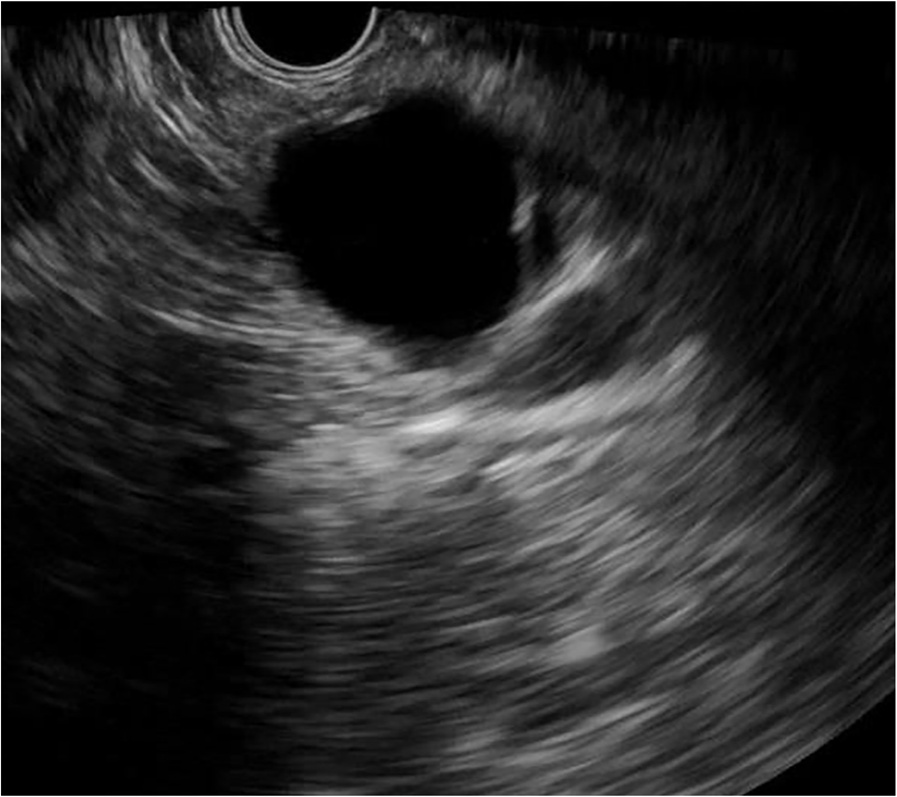
Serous cystic neoplasm with thin wall (1.8 mm)

SCNs (*n*=38) exhibited the widest range in size (11.4–100 mm) with a mean size of 45.3 mm. Notably, 18/38 of SCNs were detected in a head/neck location and 20/38 were detected in a body/tail location. SCNs were mainly lobulated (Fig. 1). A lobulated shape was observed in 21/38 SCNs, a round shape in 10/38 cases and an irregular shape (Fig. 2) in 7/38 cases. Thin walls (Fig. 3) were found in 29/38 of SCNs and thick walls were detected in 9/38 cases. More than 2 septa (Fig. 1) were present in 27/38 SCNs, while 0–2 septa were found in 11/38 cases. Solid components were rare and found in only 3/38 SCNs. □

**Fig. 4:**
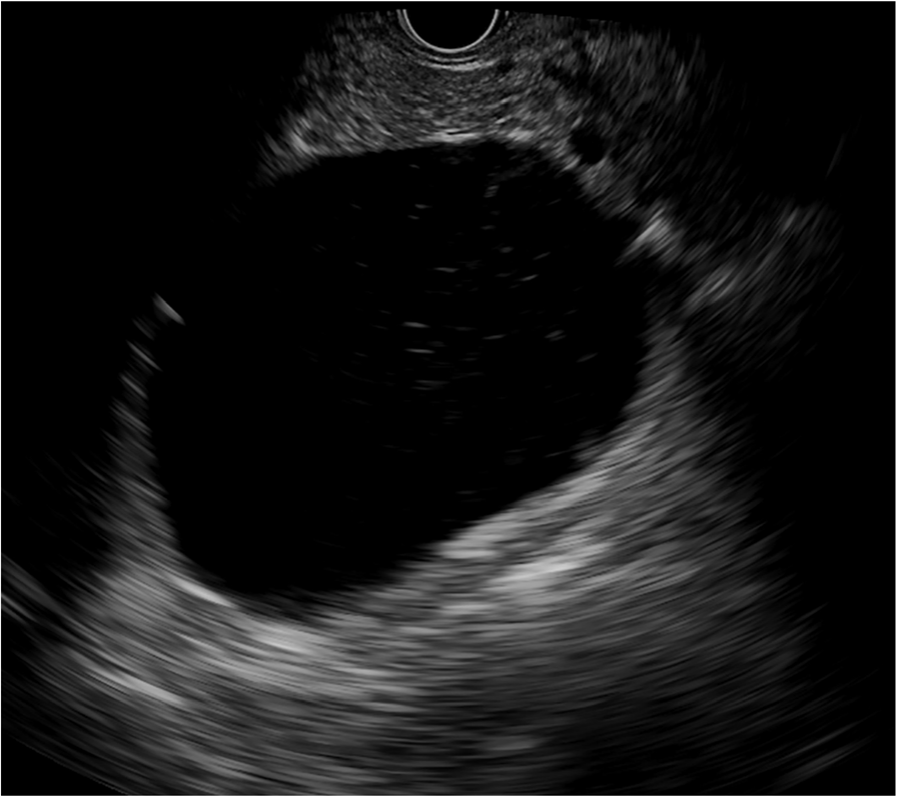
Mucinous cystic neoplasm with round shape and unilocular cyst

**Fig. 5:**
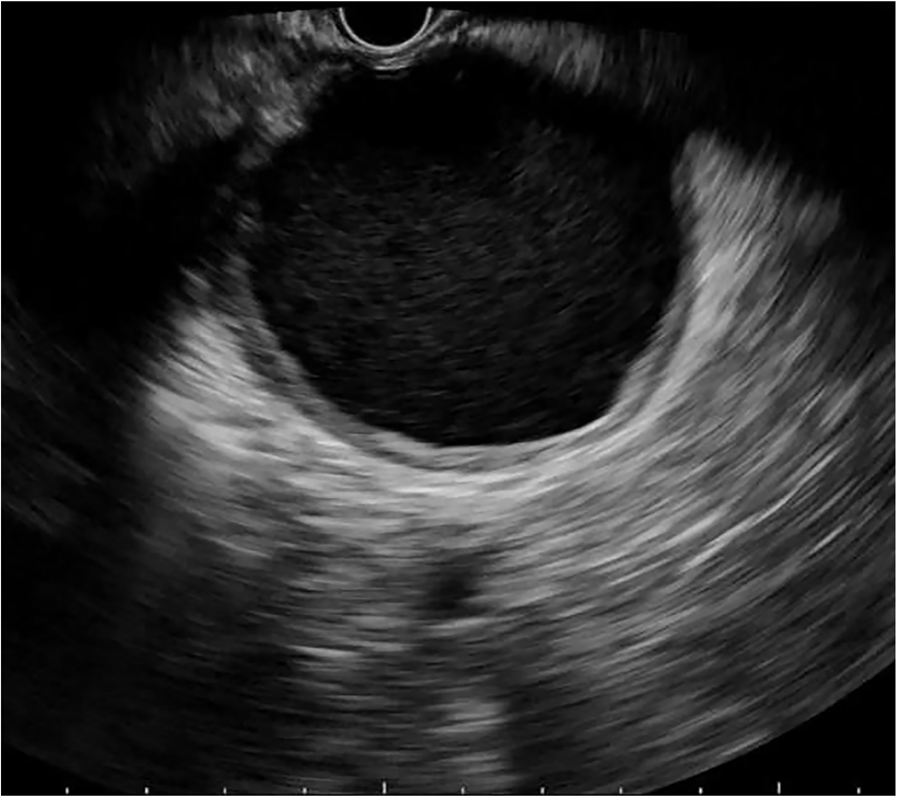
Mucinous cystic neoplasm with thick wall (3.2 mm)

**Fig. 6:**
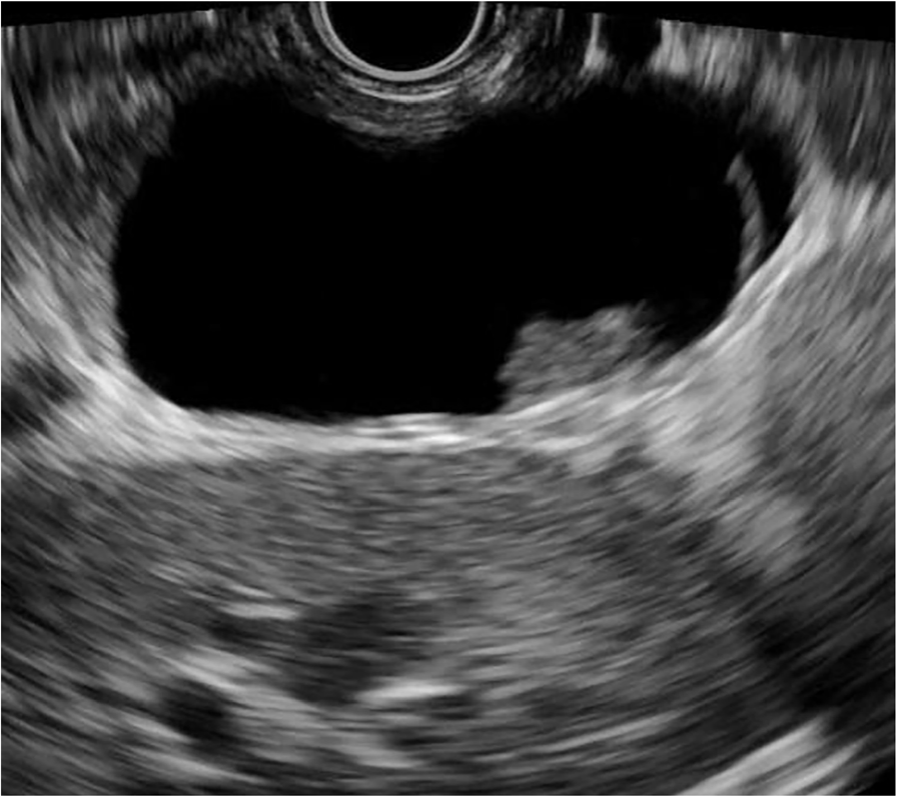
Mucinous cystic neoplasm with solid component

MCNs (*n* = 31) exhibited the widest range in size (14.8–98.8 mm) with a mean size of 49.0 mm. Additionally, 5/31 of MCNs were detected in a head/neck location and 26/31 were detected in a body/tail location. MCNs were mainly round-like (Fig. 4). A round shape was detected in 24/31 MCNs, a lobulated shape in 4/31 cases and an irregular shape in 3/31 cases. Thin walls (Fig. 5) were found in 10/31 of MCNs, and thick walls were detected in 21/31 cases. Zero to two septa (Fig. 4) were present in 20/31 MCNs, while > 2 septa were found in 11/31 cases. Solid components (Fig. 6) were found in 7/38 SCNs.

Compared with the imaging features of SCNs and MCNs, there were significant differences in the location (*P* = 0.006), shape (*P* < 0.001), cystic wall thickness (P < 0.001), and number of septa (*P* = 0.003).

In the determination of location, shape, cystic wall thickness and number of septa, there were 1 case, 3 cases, 4 cases, 2 cases with inconsistent results, respectively. The interobserver agreement was excellent. The Kappa values for identifying the four features were 0.97, 0.93, 0.88 and 0.94, respectively.

### Combination of imaging features

Table 3 presents the sensitivity, specificity, PPV, NPV and Az value of the imaging features with significant differences in Table 2 in the diagnosis of SCNs.

**Table 3:** Specificity, sensitivity, PPV, NPV and Az value of the imaging features in the diagnosis of SCNs

Imaging features	Sensitivity (95% CI)	Specificity (95% CI)	PPV (95% CI)	NPV (95% CI)	Az value (95% CI)
Head/neck location	47.4% (31.3–64.0%)	83.9% (65.5–93.9%)	78.3% (55.8–91.7%)	56.5% (41.2–70.8%)	0.656 (52.7–78.6%)
Lobulated shape	55.3% (38.5–71.0%)	87.1% (69.2–95.8%)	84.0% (63.1–94.7%)	61.3% (45.5–75.3%)	0.712 (58.9–83.5%)
Thin wall	76.3% (59.3–88.0%)	67.7% (48.5–82.7%)	74.4% (57.6–96.4%)	70.0% (50.4–84.6%)	0.720 (59.6–84.5%)
> 2 septa	71.1% (53.9–84.0%)	64.5% (45.4–80.2%)	71.1% (53.9–84.0%)	64.5% (45.3–80.2%)	0.678 (54.9–80.7%)
Two features	84.2% (68.1–93.4%)	80.6% (61.9–91.9%)	84.2% (68.1–93.4%)	80.6% (61.9–91.9%)	0.824 (71.9–93.0%)
Three features	55.3% (38.5–71.0%)	93.5% (77.2–98.9%)	91.3% (70.5–98.5%)	63.0% (47.5–76.4%)	0.744 (62.7–86.1%)
Four features	15.8% (6.6–31.9%)	100% (86.2–100%)	100% (51.7–100%)	49.2% (36.5–62.0%)	0.579 (44.5–71.3%)

*SCNs* serous cystic neoplasms, *PPV* positive predictive value, *NPV* negative predictive value, *CI* confidence interval

**Fig. 7:**
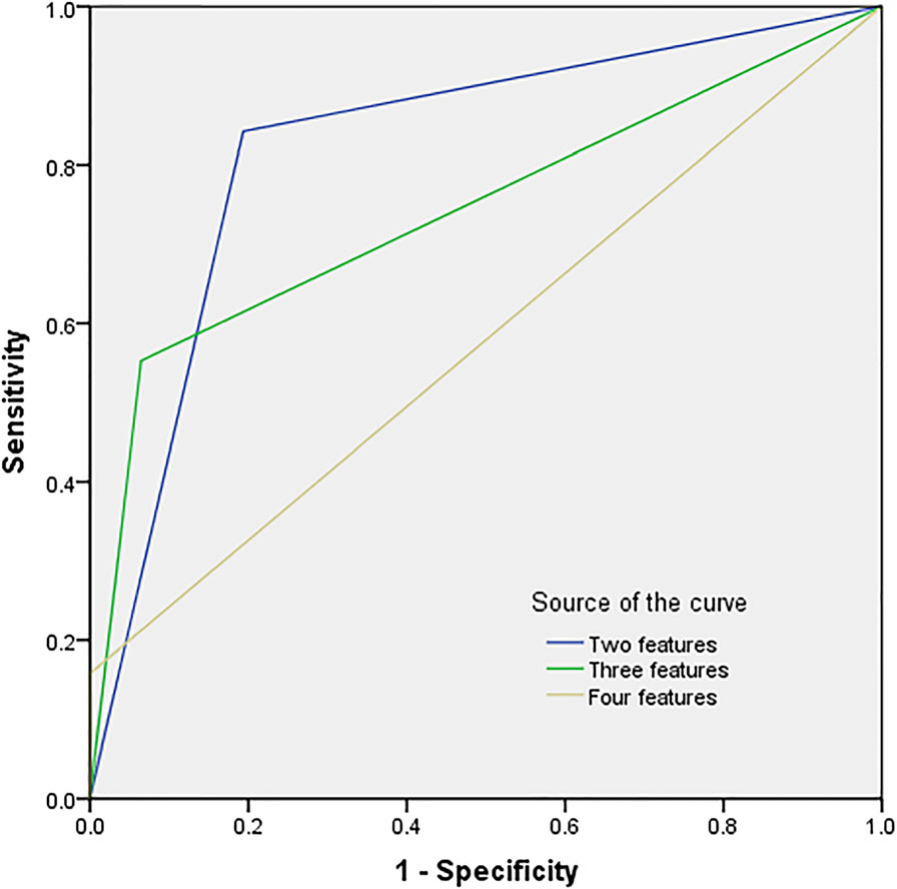
Graph shows the ROC curves of the imaging features in the diagnosis of SCNs

Head/neck location, lobulated shape, thin wall (0–2 mm) and 0–2 septa were the specific imaging features for the diagnosis of SCNs. Head/neck location and lobulated shape had high specificity (83.9, 87.1%, respectively) but low sensitivity (47.4, 55.3%, respectively). Thin wall (0–2 mm) and > 2 septa had limited sensitivity (76.3, 71.1%, respectively), specificity (67.7, 64.5%, respectively), PPV (74.4, 70.0%, respectively), and NPV (71.1, 64.5%, respectively). Among these features, only the Az value of lobulated shape and thin wall (0–2 mm) in diagnosing SCNs were greater than 0.700 (0.712, 0.720, respectively). However, when any two imaging features were combined, the sensitivity, specificity, PPV, NPV and Az values for the diagnosis of SCNs were 84.2, 80.6, 84.2, 80.6% and 0.824, respectively. When any three imaging features were combined, the sensitivity, specificity, PPV, NPV and Az values were 55.3, 93.5, 91.3, 63.0%, and 0.744, respectively. When the four imaging features were combined, the specificity was as high as 100%, the sensitivity was only 15.8%, and the Az value was also reduced to 0.579 (Fig. 7). Through comparative analysis, we determine a criterion, that is, pancreatic cystadenomas that meet any two imaging features could be diagnosed as SCNs.

Table 4 presents the sensitivity, specificity, PPV, NPV and Az value of the imaging features with significant differences in Table 2 in the diagnosis of MCNs.

**Table 4:** Specificity, sensitivity, PPV, NPV and Az value of the imaging features in the diagnosis of MCNs

Imaging features	Sensitivity (95% CI)	Specificity (95% CI)	PPV (95% CI)	NPV (95% CI)	Az value (95% CI)
Body/tail location	83.8% (65.5–93.9%)	47.4% (31.3–64.0%)	56.5% (41.2–70.8%)	78.3% (55.8–91.7%)	0.656 (52.7–78.6%)
Round shape	77.4% (58.5–89.7%)	73.7% (56.6–86.0%)	70.6% (52.3–84.3%)	80.0% (62.5–90.9%)	0.756 (63.7–87.4%)
Thick wall	67.7% (48.5–82.7%)	76.3% (59.4–88.0%)	70.0% (50.4–84.6%)	74.4% (57.6–86.4%)	0.720 (59.6–84.5%)
0–2 septa	64.5% (45.4–80.2%)	71.1% (53.9–84.0%)	64.5% (45.4–80.2%)	71.1% (53.9–84.0%)	0.678 (54.9–80.7%)
Two features	90.3% (73.1–97.4%)	60.5% (43.5–75.5%)	65.1% (49.0–78.5%)	88.5% (68.7–97.0%)	0.754 (63.8–87.1%)
Three features	77.4% (58.5–89.7%)	84.2% (68.1–93.4%)	80.0% (60.8–91.6%)	82.1% (65.9–91.9%)	0.808 (69.6–91.7%)
Four features	25.8% (12.5–44.9%)	92.1% (77.5–97.9%)	72.7% (39.3–92.7%)	60.3% (46.6–72.7%)	0.590 (45.2–72.7%)

*MCNs* mucinous cystic neoplasms, *PPV* positive predictive value, *NPV* negative predictive value, *CI* confidence interval

**Fig. 8:**
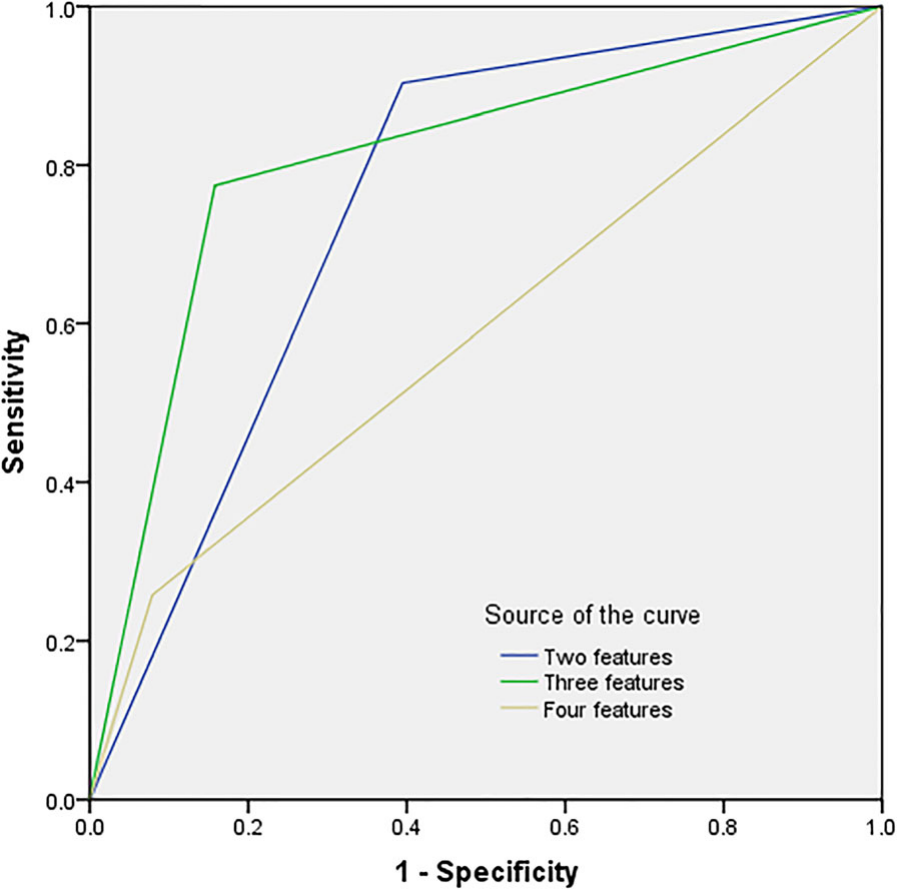
Graph shows the ROC curves of the imaging features in the diagnosis of MCNs

Body/tail location, round shape, thick wall (> 2 mm) and 0–2 septa were the specific imaging features for the diagnosis of MCNs. Body/tail location had a relatively high sensitivity (83.9%) but a low specificity (47.4%). Round shape, thick wall (> 2 mm) and 0–2 septa had similar specificity (73.7, 76.3, 70.6%, respectively) in the diagnosis of MCNs, while round shape had a relatively high Az value (0.756). The sensitivity, PPV and NPV of round shape in the diagnosis of MCNs were 77.4, 70.6, 80%. When any two imaging features were combined, the sensitivity, specificity, PPV, NPV and Az values for the diagnosis of MCNs were 90.3, 60.5, 65.1, 88.5%, and 0.754, respectively. When any three imaging features were combined, we achieved the highest Az value (0.808), with an appropriate specificity, sensitivity, PPV and NPV (77.4, 84.2, 80, 82.1%, respectively). When the four imaging features were combined, the specificity was as high as 92.1%, the sensitivity was only 25.8%, and the Az value was also reduced to 0.590 (Fig. 8). Through comparative analysis, we determine a criterion, that is, pancreatic cystadenomas that meet any three imaging features could be considered as MCNs.

## Discussion

PCLs can be divided into neoplastic cystic lesions and nonneoplastic cystic lesions. Nonneoplastic cystic lesions mainly refer to pseudocysts. Neoplastic cystic lesions mainly include SCNs, MCNs, intraductal papillary mucinous neoplasms (IPMNs) and cystic degeneration of solid tumors. The pancreatic solid-cystic tumors are rare and relatively easy to diagnose. Therefore, it is crucial to differentiate SCNs and MCNs from pseudocysts and IPMNs. Pseudocysts mostly form after inflammation, necrosis or hemorrhage related to pancreatitis or trauma, and are enclosed by a wall with fibrous tissue. Pseudocysts mainly manifest as round cysts, generally without septa [11]. IPMNs can be classified as main duct IPMNs (MD-IPMNs) and branch duct IPMNs (BD-IPMNs). The MD-IMPNs are characterized by segmental or diffuse dilatation of the main pancreatic duct, which may resemble chronic pancreatitis [12]. The BD-IPMNs are composed of cysts that communicate with the main pancreatic duct [12]. Both IPMN and pseudocysts are predominant in men and are prone to pancreatitis [13, 14]. Our study revealed that SCNs and MCNs occurred more frequently in women, which was consistent with previous studies. It was relatively simple to differentiate pseudocysts and IPMNs from SCNs and MCNs.

SCNs have different biological characteristics than MCNs. SCNs are generally benign, with only 1 to 3% malignant potential [3], and can be followed up [5]. Approximately 10% of SCNs are manifested as unilocular without septa, which are easily misdiagnosed as MCNs [13]. MCNs have malignant potential and are recommended for surgical resection once an adequate diagnosis has been performed [6, 7]. Therefore, it is of great significance to correctly differentiate between SCNs and MCNs for the appropriate treatment. In 2005, Sahani et al. [15] proposed a simple imaging-based classification system for guiding the management of PCLs. PCLs were classified into unilocular cysts, microcystic lesions, macrocystic lesions and cysts with a solid component. However, this classification system did not effectively identify SCNs and MCNs. In 2017, Zhang WG et al. [16] first proposed a new criterion to differentiate between SCNs and MCNs by EUS. This study enrolled only 41 patients diagnosed with SCNs and MCNs. The sample size included was limited. At present, there is no uniform standard for the differential diagnosis of SCNs and MCNs, and it is still difficult to accurately differentiate between SCNs and MCNs.

In our study, SCNs and MCNs were predisposed to occur in middle-aged women (49.16 years vs 46.39 years), which was slightly different from previous studies. Relevant studies have demonstrated that MCNs occurred almost exclusively in women (> 98%) and were generally diagnosed in patients in their 40s and 50s [17, 18], while SCNs occurred more commonly in women, who typically presented in their 60s [19]. The difference may be related to the insufficient sample size in our study.

SCNs and MCNs have different histological characteristics. The location distribution of SCNs was different from that of MCNs. MCNs were prone to occur in the body/tail location, and SCNs were more likely to occur in the head/neck location, which was in accordance with previous literature [20, 21]. SCNs are mainly lobulated, while MCNs are mainly round-like [22]. Multiple thin septa can be detected in SCNs. According to the number and size of daughter cysts, SCNs can be classified as microcystic, mixed macrocystic and microcystic, macrocystic, and solid types. Microcystic SCNs are composed of multiple cysts of varying sizes, from a few millimeters up to two centimeters. Macrocystic SCNs are characterized by a predominantly or exclusively unilocular pattern [23], which is also called an “oligo-cystic pattern”. Regarding septa, the “oligo-cystic pattern” refers to the 0–2 septa pattern. Previous studies have demonstrated that microcystic SCNs accounted for approximately 45%, while macrocystic SCNs accounted for approximately 32% [24], which was similar to our results. MCNs usually present as round macrocystic lesions with no or few septa [12]. The cystic wall of SCNs is thinner than that of MCNs. Khurana B et al. [25] revealed that the cystic wall was less than 2 mm thick in four (80%) of the five SCNs.

Head/neck location, lobulated shape, thin wall (0–2 mm) and > 2 septa were the specific imaging features in the diagnosis of SCNs. The Az value of a single imaging feature in the diagnosis of SCNs is not ideal. When any two imaging features were combined, we obtained the highest Az value (0.824) in diagnosing SCNs, as well as the appropriate sensitivity (84.2%), specificity (80.6%), PPV (84.2%), NPV(80.6%) which was almost consistent with study by Sun Y [14]. The four specific imaging features used in the study by Sun Y [14] for the diagnosis of SCNs were head/neck location, lobulated shape, thin wall (0–2 mm) and honeycomb pattern, which were slightly different from the imaging features used in our study. We believed that the honeycomb pattern was more specific for the diagnosis of SCNs than the number of septa > 2 but excluded a large portion of atypical SCNs. In our study, > 2 septa were eventually used as one of the four EUS image features, and we achieved good diagnostic efficacy.

Body/tail location, round shape, thick wall and 0–2 septa were the specific imaging features for the diagnosis of MCNs. Compared with any other single imaging signs in the diagnosis of MCNs, round shape had the highest Az value (0.756), with a relatively appropriate sensitivity (77.4%), specificity (73.7%), PPV (70.6%) and NPV (80.0%). When the imaging features were combined, the combined diagnosis of any three features could obtain the highest Az value (0.808), as well as the appropriate sensitivity (77.4%), specificity (84.2%), PPV (80.0%) and NPV (82.1%), which was almost consistent with the results reported by Sun Y [14]. The combined diagnosis of any three features was more advantageous than the round shape feature in the diagnosis of MCNs.

There are three limitations in our research. First, the sample size is not large enough; second, the criteria are only applicable to the differential diagnosis of SCNs and MCNs, but not to the diagnosis of other PCLs; third, due to the limited sample size, we did not conduct a comparative study of macrocystic SCNs and MCNs.

## Conclusion

Head/neck location, lobulated shape, thin wall (0–2 mm) and > 2 septa were the specific imaging features for the diagnosis of SCNs. Pancreatic cystadenomas that meet any two imaging features could be diagnosed as SCNs.

Body/tail location, round shape, thick wall (> 2 mm) and 0–2 septa were the specific imaging features for the diagnosis of MCNs. Pancreatic cystadenomas that meet any three imaging features could be considered as MCNs.

## Data Availability

All datasets used and analyzed during the current study are available from the corresponding author upon reasonable request.

## Abbreviations

BD-IPMNs: branch duct IPMNs
CI: confidence interval
CT: computed tomography
EUS: endoscopic ultrasound
IPMNs: intraductal papillary mucinous neoplasms
MCNs: mucinous cystic neoplasms
MD-IPMNs: main duct IPMNs
MRI: magnetic resonance imaging
NPV: negative predictive value
PCLs: pancreatic cystic lesions
PCNs: pancreatic cystic neoplasms
PPV: positive predictive value
SCNs: serous cystic neoplasms
